# Cysticercosis Involving Muscle of Mastication: A Review and Report of Two Cases

**DOI:** 10.1155/2013/814126

**Published:** 2013-12-15

**Authors:** Sarbjeet Singh, V. Sreenivasan, Kanika Garg, Nikhel Dev Wazir, Jaspal Singh Rajput, Pawandeep Sandhu Virk

**Affiliations:** ^1^Department of Oral Medicine & Radiology, Institute of Dental Sciences, Sehora, Jammu 181132, India; ^2^Department of Oral Medicine & Radiology, Subharti Dental College and Hospital, Meerut, Uttarpradesh 250002, India; ^3^Department of Conservative & Endodontics, Institute of Dental Sciences, Sehora, Jammu 181132, India; ^4^Department of Pedodontics, SGT Dental College, Hospital & Research Center, Gurgoan, Haryana 122505, India; ^5^Department of Pedodontics, Desh Bhagat Dental College & Hospital, Muktsar, Punjab 152026, India

## Abstract

Cysticercosis is a parasitic infection caused by the larval stages of the parasitic cestode, *Taenia solium*. It is a common disease in developing countries where it is also endemic. The central nervous system (CNS) is the most important primary site of infection and the disease can present with solitary or multiple space occupying lesions. Cases of cysticercosis presenting as isolated muscle mass (pseudotumours) without involvement of the CNS have also been recently described in the literature. We present two cases who presented to us with pain, swelling, and tenderness involving the temporalis muscle along with trismus. Ultrasonography and MRI findings were suggestive of cysticercosis involving the temporalis muscle which resolved after the albendazole therapy.

## 1. Introduction

Cysticerci are spherical milky white cysts containing fluid and a single invaginated scolex with hooklets [1]. When humans ingest eggs or gravid proglottids from the parasite *Taenia solium*, the covering of the eggs is digested in the stomach and the larval form (cysticercus cellulosae) of the parasite is hatched [1]. The larvae penetrate the mucosa, enters the blood vessels and lymphatics, and are distributed in the tissues all over the body but preferentially locate in the brain, muscle, skin, liver, lungs, and heart [1]. They are also found in oral and perioral tissues, particularly in the muscles of mastication, facial expression, the suprahyoid muscles, and the postcervical musculature as well as in the tongue, buccal mucosa, and lip [2–4]. Here we report two cases of cysticercosis affecting the temporalis muscle.

### 1.1. Case Report 1

A 50-year-old female patient presented to our department with complaint of pain and mild swelling on the right side of the face since the last 2 months. She gave a history that pain is aggravated while opening the mouth, chewing and on application of pressure on that area and was not relieved by NSAIDs. Her past medical history revealed history of typhoid fever 3 months back for which she had taken complete course of treatment.

**Figure 1:**
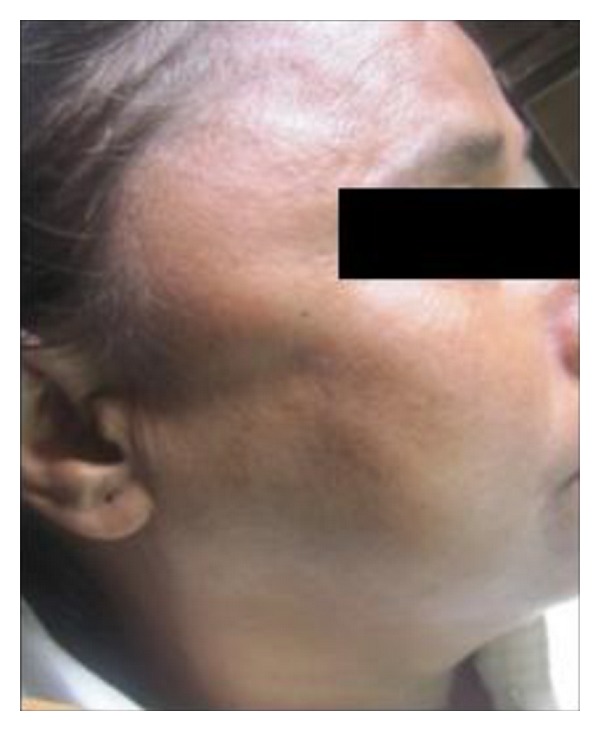


On extraoral examination, maximal mouth opening was reduced to around 28 mm and a diffuse swelling around 1 × 1 cms in diameter was also observed in the right temporalis muscle which was soft to firm in consistency and was tender on palpation (Figure 1). Examination of the TMJ and other muscles of mastication revealed normal findings. On intraoral examination edentulous spaces were present involving the right and left mandibular posterior teeth.

**Figure 2:**
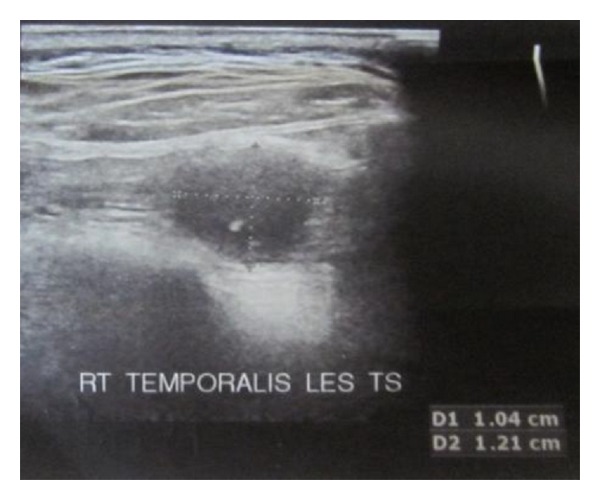


**Figure 3:**
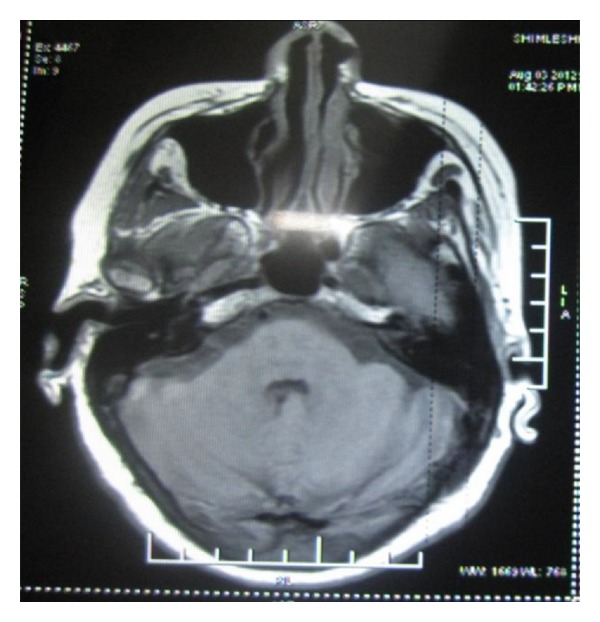


On the basis of the history and examination findings, a clinical provisional diagnosis of myofascial pain was made and the patient was put on the treatment of muscle relaxants Tab Myospaz forte two times a day and topical application of voveran emuigel along with hot fomentation, soft diet, and bilateral chewing. The patient reported to us after 3 days but she did not have relief whatsoever. Reevaluation of the case was done and she was advised of blood investigations along with ultrasonogram (USG) and MRI involving the right side of the face. The findings of the blood investigations were normal. USG showed the presence of a well-defined anechoic lesion around 1.2 × 1 × 0.9 cms involving the right temporalis muscle with its long axis parallel to its fibres and 14 × 12 mm echogenic nidus was also seen within it (Figure 2). MRI revealed the presence of a rounded iso to mildly hyperintense lesion in the right temporalis muscle with an identifiable hypointense nidus on T1 and T2 and Fiesta-C images (Figure 3). The lesion also displayed T1 and T2 hypointense rim along with edema involving the right temporalis muscle. The features on both USG and MRI were suggestive of cysticercosis involving the right temporalis muscle.

**Figure 4:**
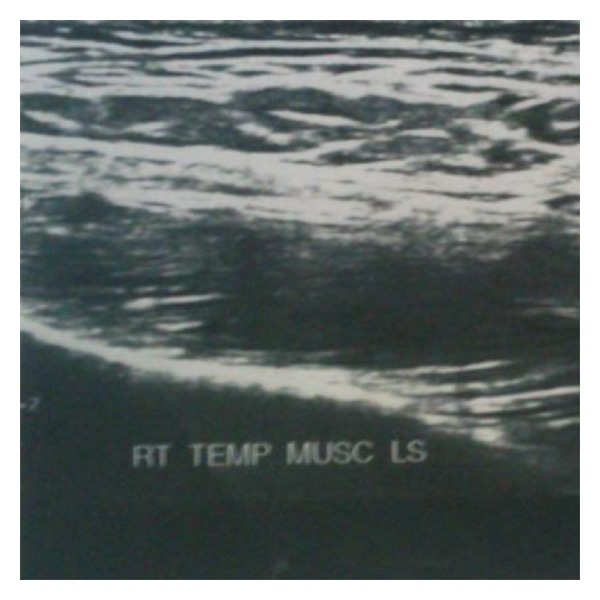


**Figure 5:**
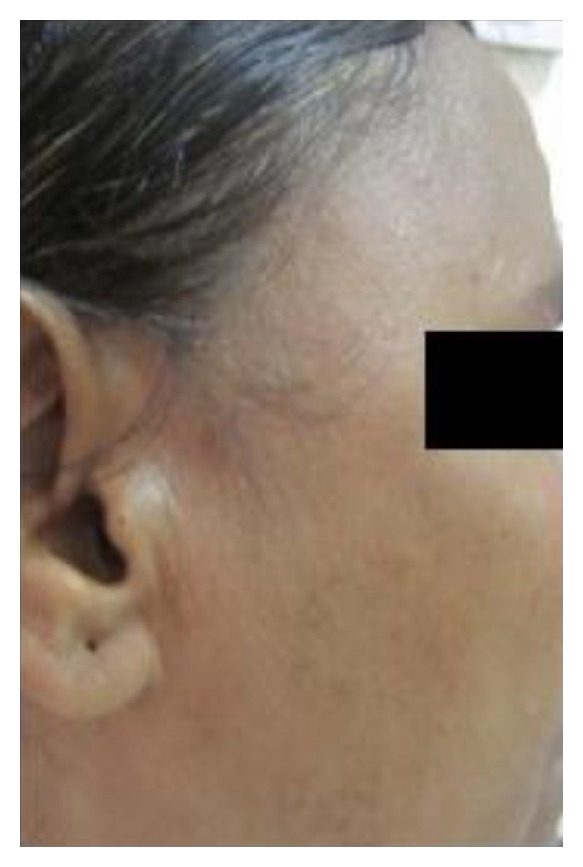


For the treatment the patient was advised of anthelmintic albendazole 400 mg BD AND Crocin SOS for 4 weeks. Four-week follow-up of the patient showed significant reduction in the pain and the size of the swelling. Mild swelling stiltreatment was continued for another 5 days. USG examination after 4 weeks and 5 days showed coml persisted so the plete resolution of the lesion with no focal lesion identifiable in the right temporalis muscle (Figure 4). The swelling and pain had also subsided completely (Figure 5).

### 1.2. Case Report 2

**Figure 6:**
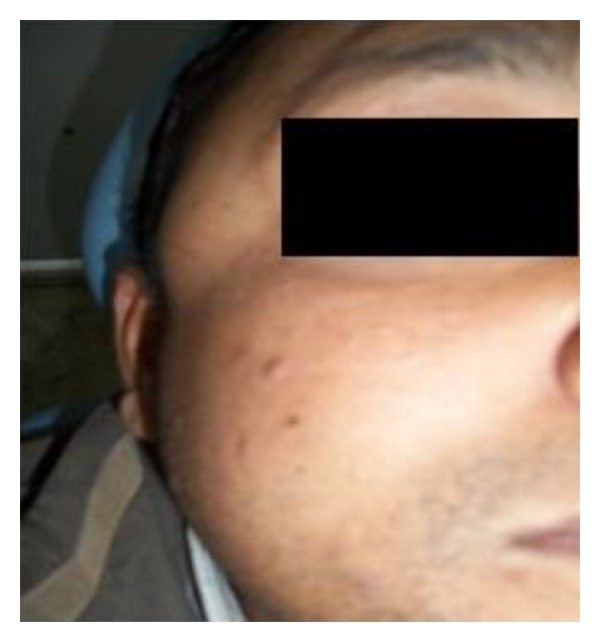


**Figure 7:**
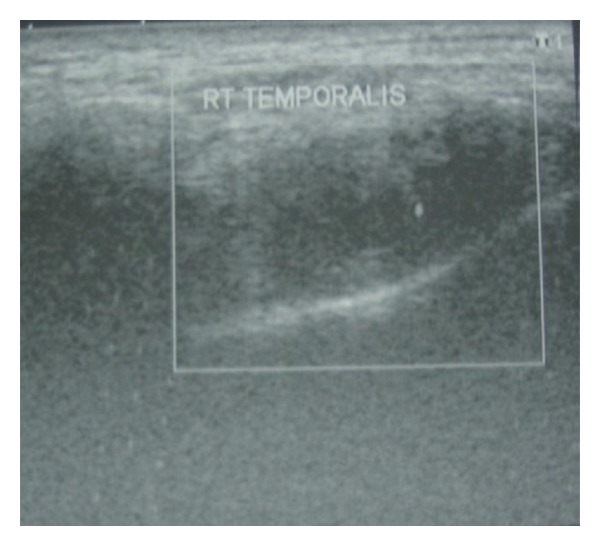


**Figure 8:**
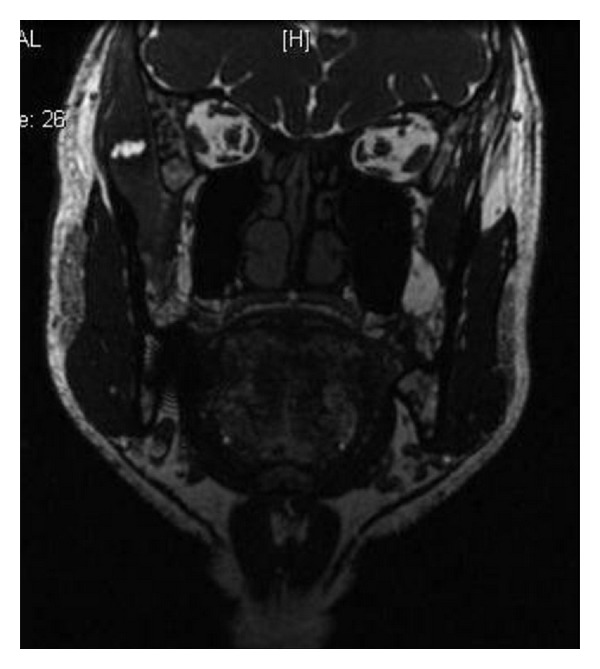


A 42-year-old man complained of a gradually increasing swelling over the right temple region. The patient also complained of associated difficulty in speaking or chewing food. On examination, there was a well-defined, firm, and tender swelling of 2 × 1.5 cm involving the temporalis muscle on the right side (Figure 6). This time the patient was provisionally diagnosed as cysticercosis involving the temporalis muscle. A high-resolution sonographic examination of the temporal swelling was done. Sonography showed a well-defined, 1.5 × 1.8 cm, hypoechoic lesion with a small hyperechoic speck suggesting a scolex (Figure 7). Hence, the possibility of cysticercosis was considered. MRI (Figure 8) revealed the presence of a rounded hyperintense lesion in the right temporalis muscle with an identifiable hypointense nidus on T1 and T2 and Fiesta-C images. The features on both USG and MRI were suggestive of cysticercosis involving the right temporalis muscle. The patient was prescribed Tab albendazole 400 mg BD along with Crocin SOS for 4 weeks. The patient completely recovered and was completely asymptomatic after 4 weeks.

## 2. Discussion

Tapeworm infection is common in developing countries where the combination of rural society, crowding, and poor sanitation allows greater contact between humans and pigs and thus more opportunities for fecal contamination of food and water [1, 2].

Normally, humans are the definitive hosts for *T. solium*, the life cycle of which begins with ingestion of viable larvae in inadequately cooked pork. The cyst wall is destroyed by gastric secretion, releasing 1 scolex that passes into the small intestine, where it becomes fixed. Embryonated eggs and gravid proglottids are released in the feces, deposited on the soil, and later ingested by the intermediate host, the pig. Ingestion of unwashed green leafy vegetables like cabbage is also one of the sources of infection. The animal's gastric secretions destroy the egg wall, and after the passage into the duodenum, the larvae release from the eggs, penetrate the intestinal wall, and are carried by blood or lymph to various tissues. In the pig, the muscle tissue is particularly involved. Once in the muscle, the larvae develop into cysticerci [4]. The cystic structure contains a small, invaginated scolex and neck resembling the adult form. Ingestion of undercooked pork by humans once again initiates the parasitic cycle. If humans ingest eggs from adult tapeworm segments passed in human feces, they can become intermediate hosts. Humans can acquire the infection by ingesting water or food contaminated with human feces, by oral transmission via the hands of carriers of adult worms, or by internal regurgitation of eggs into the stomach after reverse peristalsis. The clinical course of cysticercosis depends on the number of cysts, the particular tissue infected, and the reaction of tissues to the organism [3]. The larvae may migrate to any organ and may remain viable for many years [4]. The organism most often invades the central nervous system, eye, subcutaneous tissue, skeletal muscle, and heart, but occasionally the lungs, liver, and kidneys may be affected. Skeletal involvement may cause transient tenderness and either muscular atrophy or hypertrophy [3].

The literature review of the cases involving cysticercosis affecting muscles of mastication shows just two reports of cysticercosis affecting temporalis muscle and 5 case reports affecting the masseter muscle (Table 1).

All the cases presented with muscle tenderness and swelling similar to our cases. Diagnostic tests include radiologic imaging (particularly computerized tomography and MRI), serology, and tissue biopsy. Medical treatment with Albendazole and praziquantel (50 mg·kg^−1^·d^−1^ in 3 divided doses for 14 days and 75 mg·kg^−1^·d^−1^ for 10 days) has been recommended for neurocysticercosis and subcutaneous cysticercosis [8]; however, praziquantel has no effect on calcified parasites. Preventive measures are important and include the thorough cooking of pork and all vegetables and early detection and complete removal of the worm.

## Figures and Tables

**Table 1:** 

	Author/year	Muscles affected	No. of cases
(1)	Reddi et al. [5]/2001	Masseter	1
(2)	Sidhu et al. [6]/2002	Masseter	1
(3)	Sethi et al. [7]/2007	Temporalis	1
(4)	Mittal et al. [8]/2008	Masseter	1
(5)	Gokarn et al. [9]/2011	Masseter	1
(6)	Kumar et al. [10]/2011	Temporalis	1
(7)	Kumar et al. [11]/2011	Masseter	1
(8)	Present cases	Temporalis	2
